# Retraction: Molecular prevalence and phylogeny of *Anaplasma marginale*, *Anaplasma ovis* and *Theileria ovis* in goats and sheep enrolled from a hill station in Punjab, Pakistan

**DOI:** 10.1371/journal.pone.0358284

**Published:** 2026-09-14

**Authors:** The PLOS One Editors

The *PLOS One* Editors retract this article [1] because it was identified as one of a series of submissions for which the peer review process was compromised and there are concerns about compliance with PLOS policy on Manipulation of the Publication Process.

SS, NN, AK, NM, and FI did not agree with the retraction. MAr, AB, MF, MAs, MAK, MI, HM, and CCC either did not respond directly or could not be reached.
